# Supplementary material for: Aorto-Iliac Artery Calcification Prior to Kidney Transplantation
Source: J Clin Med. 2020 Sep 7;9(9):2893. doi: 10.3390/jcm9092893 (PMC7563260; doi:10.3390/jcm9092893)
Supplement: Supplementary file 1 [file jcm-09-02893-s001.pdf]

Article

Aorto-Iliac Artery Calcification Prior to Kidney Transplantation

Stan Benjamens [1,2]*, Elsaline Rijkse [3], Charlotte A. te Velde-Keyzer [4], Stefan P. Berger [4], Cyril Moers [1],

Martin H. de Borst [4], Derya Yakar [2], Riemer H. J. A. Slart [2,5], Frank J. M. F. Dor [6,7], Robert C. Minnee [3] and Robert A. Pol [1]

[1] Department of Surgery, Division of Transplant Surgery, University of Groningen, University Medical Center Groningen, Groningen, The Netherlands; c.moers@umcg.nl (C.M.); r.pol@umcg.nl (R.A.P.)

[2] Medical Imaging Center, Department of Nuclear Medicine and Molecular Imaging & Department of Radiology, University of Groningen, University Medical Center Groningen, Groningen, The Netherlands; d.yakar@umcg.nl (D.Y.); r.h.j.a.slart@umcg.nl (R.H.J.A.S.)

[3] Department of Surgery, Division of HPB and Transplant Surgery, Erasmus MC University Medical Center, Rotterdam, The Netherlands; a.rijkse@erasmusmc.nl (E.R.); r.minnee@erasmusmc.nl (R.B.M.)

[4] Department of Internal Medicine, Division of Nephrology, University of Groningen, University Medical Center Groningen, Groningen, The Netherlands; c.a.keyzer@umcg.nl (C.A.t.V.K.); s.p.berger@umcg.nl (S.P.B.); m.h.de.borst@umcg.nl (M.H.d.B)

[5] Department of Biomedical Photonic Imaging, Faculty of Science and Technology, University of Twente, Enschede, The Netherlands.

[6] Imperial College Renal and Transplant Centre, Hammersmith Hospital, Imperial College Healthcare NHS Trust, London, United Kingdom; frank.dor@nhs.net (F.J.M.F.D.)

[7] Department of Surgery & Cancer, Imperial College, London, United Kingdom

* Correspondence: s.benjamens@umcg.nl (S.B.)

Table 1. Univariable associations with all-cause mortality.

**Covariables**	**Hazard ratio**	**95% CI**	***p*-value**
CaScore (stratified)			
Low	Ref.	Ref.	
Medium	2.12	1.08-4.15	**0.029**
High	5.24	2.85-9.63	**<0.0001**
CaScore (continuous)	2.02	1.60-2.56	**<0.0001**
Female gender	0.96	0.61-1.51	0.855
Age	1.06	1.03-1.08	**<0.0001**
Diabetes mellitus	2.23	1.43-3.49	**<0.001**
Body Mass Index	1.01	0.96-1.06	0.713
Smoker			
Non	Ref.	Ref.	
Former	1.21	0.71-2.05	0.489
Current	2.27	1.26-4.08	**0.006**
Total cholesterol	1.18	1.00-1.39	0.052
Hypercholesterolemia	1.45	0.92-2.27	0.108
Systolic blood pressure	1.01	1.00-1.02	0.057
Diastolic blood pressure	0.98	0.97-1.00	**0.033**
Use of antihypertensive medication	1.97	0.99-3.95	0.055
Type of dialysis			
Pre-emptive	Ref.	Ref.	
Hemodialysis	2.94	1.59-5.42	**<0.001**
Peritoneal dialysis	2.19	1.07-4.48	**0.032**
Dialysis vintage pre-transplantation	1.34	1.15-1.56	**<0.001**
History of MI	1.76	1.04-2.98	**0.035**
History of CVA	1.65	0.80-3.44	0.177
History of TIA	0.33	0.05-2.40	0.276
History of PAD	2.55	1.53-4.24	**<0.001**
Charlson Comorbidity Index	1.25	1.16-1.35	**<0.0001**
Framingham score	1.86	1.43-2.42	**<0.0001**

Aorto-iliac calcium score = CaScore; hazard ratio, 95% confidence interval (95% CI), and *p*-value by Cox proportional hazards regression analysis; MI = myocardial infarction; CVA = cerebrovascular accident; TIA = transient ischemic attack; PAD = new onset peripheral artery disease.

Table S2. Univariable Cox regression analysis for cardiovascular mortality.

Covariables	Hazard ratio	95% CI	*p*-value
CaScore			
Low	Ref.	Ref.	
Medium	3.42	1.07-10.91	**0.038**
High	7.86	2.63-23.47	**<0.001**
CaScore (continuous)	2.27	1.55-3.34	**<0.0001**
Female gender	1.16	0.57-2.36	0.672
Age	1.07	1.03-1.11	**<.001**
Diabetes mellitus	2.42	1.20-4.90	**0.014**
Body Mass Index	1.03	0.96-1.11	0.361
Smoker			
Non	Ref.	Ref.	
Former	4.03	1.38-11.45	**0.011**
Current	2.80	0.82-9.57	.102
Total cholesterol	1.41	1.11-1.79	**0.005**
Hypercholesterolemia	1.79	0.89-3.60	0.103
Systolic blood pressure	1.00	0.99-1.02	0.955
Diastolic blood pressure	0.97	0.94-0.99	**0.014**
Use of antihypertensive medication	3.76	0.90-15.70	0.070
Type of dialysis			
Pre-emptive	Ref.	Ref.	
Hemodialysis	0.94	0.41-2.18	0.894
Peritoneal dialysis	2.17	0.90-5.25	0.086
Dialysis vintage pre-transplantation	1.47	1.13-1.90	**0.004**
History of MI	2.83	1.33-5.98	**0.007**
History of CVA	1.00	0.24-4.21	0.996
History of TIA	0.87	0.12-6.35	0.887
History of PAD	2.57	1.15-5.75	**0.021**
Charlson Comorbidity Index	1.23	1.08-1.40	**0.002**
Framingham score	1.93	1.26-2.96	**0.002**

Aorto-iliac calcium score = CaScore; hazard ratio, 95% confidence interval (95% CI), and *p*-value by Cox proportional hazards regression analysis; MI = myocardial infarction; CVA = cerebrovascular accident; TIA = transient ischemic attack; PAD = new onset peripheral artery disease.

Table S3. Univariable Cox regression analysis for cardiovascular events.

**Covariables**	**Hazard ratio**	**95% CI**	***p*-value**
CaScore			
Low	Ref.	Ref.	
Medium	2.13	1.19-3.84	**0.011**
High	2.86	1.62-5.08	**<0.001**
CaScore (continuous)	1.56	1.25-1.95	**<0.0001**
Female gender	0.93	0.60-1.45	0.757
Age	1.04	1.02-1.06	**<0.001**
Diabetes mellitus	1.94	1.26-3.01	**0.003**
Body Mass Index	1.06	1.01-1.10	**0.014**
Smoker			
Non	Ref.	Ref.	
Former	1.03	0.64-1.67	0.890
Current	1.02	0.49-1.97	0.962
Total cholesterol	1.11	0.94-1.31	0.214
Hypercholesterolemia	2.66	1.73-4.10	**<0.0001**
Systolic blood pressure	1.01	1.00-1.02	0.162
Diastolic blood pressure	0.98	0.97-1.00	0.057
Use of antihypertensive medication	1.34	0.74-2.42	0.337
Type of dialysis			
Pre-emptive	Ref.	Ref.	
Hemodialysis	2.95	1.64-5.33	**<0.001**
Peritoneal dialysis	2.17	1.08-4.38	**0.030**
Dialysis vintage pre-transplantation	1.31	1.13-1.51	**<0.001**
History of MI	3.39	2.15-5.37	**<0.0001**
History of CVA	1.94	1.00-3.77	**0.049**
History of TIA	1.98	0.86-4.56	0.107
History of PAD	1.21	0.66-2.24	0.536
Charlson Comorbidity Index	1.16	1.07-1.26	**<0.001**
Framingham score	1.54	1.21-1.96	**<0.001**

Aorto-iliac calcium score = CaScore; hazard ratio, 95% confidence interval (95% CI), and *p*-value by Cox proportional hazards regression analysis; MI = myocardial infarction; CVA = cerebrovascular accident; TIA = transient ischemic attack; PAD = new onset peripheral artery disease.

Table S4. Discriminative capacity of the CaScore for risk prediction.

	C-statistics	95% CI	Change	95% CI	*p*-value	IDI (%)	*p*-value
**All-cause mortality**							
CaScore	0.66	0.59-0.72	-	-	-	-	-
Age and gender	0.67	0.57-0.76	Ref	-	-	Ref	-
Age and gender + CaScore	0.74	0.65-0.83	0.07	0.01-0.14	**.042**	4.1	**<0.0001**
Charlson Comorbidity Index	0.71	0.64-0.78	Ref	-	-	Ref	-
Charlson Comorbidity Index + CaScore	0.75	0.67-0.83	0.04	-0.01-0.10	.131	4.2	**<0.0001**
Framingham score	0.72	0.65-0.79	Ref	-	-	Ref	-
Framingham score + CaScore	0.76	0.69-0.83	0.04	-0.04-0.13	.305	2.5	**<0.001**
**Cardiovascular mortality**							
CaScore	0.72	0.63-0.81	-	-	-	-	-
Age and gender	0.66	0.56-0.75	Ref	-	-	Ref	-
Age and gender + CaScore	0.71	0.62-0.81	0.06	-0.01-0.12	.080	1.5	**0.025**
Charlson Comorbidity Index	0.70	0.63-0.78	Ref	-	-	Ref	-
Charlson Comorbidity Index + CaScore	0.73	0.65-0.81	0.03	-0.07-0.13	.594	2.2	**0.002**
Framingham score	0.66	0.75-0.75	Ref	-	-	Ref	-
Framingham score + CaScore	0.72	0.65-0.80	0.06	-0.04-0.17	.229	1.1	**0.033**
**Cardiovascular events**							
CaScore	0.58	0.52-0.64	-	-	-	-	-
Age and gender	0.58	0.52-0.65	Ref	-	-	Ref	-
Age and gender + CaScore	0.58	0.53-0.64	0.01	-0.04-0.05	.834	1.2	**0.016**
Charlson Comorbidity Index	0.61	0.55-0.67	Ref	-	-	Ref	-
Charlson Comorbidity Index + CaScore	0.62	0.57-0.69	0.02	-0.03-0.06	.491	1.1	**0.021**
Framingham score	0.59	0.53-0.65	Ref	-	-	Ref	-
Framingham score + CaScore	0.59	0.53-0.65	0.00	-0.04-0.04	.949	0.5	0.129

Aorto-iliac calcium score = CaScore; data are presented as Harrell's concordance index (C-statistic) with 95% confidence interval (CI). Change in C-statistics and Integrated discrimination improvement (IDI) compared to model excluding CaScore, with corresponding *p*-values.
